# Real World Case Series: A.G.E. Interrupter Ultra Serum

**DOI:** 10.1111/jocd.70302

**Published:** 2025-07-25

**Authors:** Sonya Abdulla, Patricia Brieva, Hugues Cartier, Christine Dierickx, Sophie Guénin, Ariel Haus, Jani van Loghem, Ali Shahbaz, Nikolaus Schicher, Peter Bjerring

**Affiliations:** ^1^ Dermatology on Bloor Toronto Ontario Canada; ^2^ SkinCeuticals New York New York USA; ^3^ Clinic Saint‐Jean Arras France; ^4^ SkinPeriu III Bertrange Luxembourg; ^5^ Mount Sinai Dermatology New York New York USA; ^6^ Dr Haus Dermatology London UK; ^7^ Department of Dermatology University of Amsterdam Amsterdam the Netherlands; ^8^ Westlake Dermatology and Cosmetic Surgery Austin Texas USA; ^9^ Dr. Nikolaus Schicher Dermatology Klagenfurt Austria; ^10^ Department of Dermatology Aalborg University Hospital Aalborg Denmark

**Keywords:** aging face, cosmeceuticals, mature skin

## Abstract

**Background:**

The A.G.E. Interrupter serum has been developed in an oil‐in‐water emulsion to contain 30% proxylane, *Cassia alata* extract, blueberry and pomegranate extract, rhamnose, and gentiana lutea root extract. The technology has several patent ingredients as well as synergistic patents with proxylane, rhamnose, and new ones submitted for the new ingredients. An ex vivo model using this final formulation has shown upregulation of laminin‐5, collagen IV, tropoelastin, and collagen III. Collagen IV and laminin are present in the dermal‐epidermal junction (DEJ) and are factors in the basement membrane function. Over time, the DEJ decreases in height and in surface area, leading to weakened skin integrity and structure. The serum was tested to illustrate visible life in the jawline, forehead, cheek, and neck as well as instrumental testing showing visible improvement in the nasolabial fold and forehead wrinkle.

**Aim:**

To present a real‐world case series that demonstrates the use of the A.G.E. Interrupter serum as an adjunctive therapy to collagen‐stimulating procedures in a wide variety of patient ages and skin types.

**Methods:**

Eight internationally recognized, licensed dermatologists with extensive experience in cosmetic and facial rejuvenation procedures were selected to participate in an expert panel. During the expert panel, dermatologists were asked to share 2 cases using the A.G.E. Interrupter serum in patients undergoing collagen‐stimulating procedures.

**Results:**

Following the panel discussion, seven patient cases were selected to illustrate the use of the A.G.E. Interrupter serum in conjunction with collagen‐stimulating procedures to optimize patient outcomes and satisfaction. Experts found that daily A.G.E. Interrupter serum application post‐procedure led to improved overall results and high patient satisfaction.

**Conclusion:**

This case series provides evidence for different integrated skincare regimens using the A.G.E. Interrupter serum in a variety of patients.

## 1 Introduction

Collagen and its triple‐helix structure serves as a key scaffold protein in skin structure. It provides skin with mechanical strength, elasticity, and confers skin with biochemical properties that draw in hydration and maintain skin smoothness and firmness [1]. There are 28 types of collagen amongst which type I and type III are the predominant collagens in skin architecture [1]. Collagen is produced by fibroblasts in the dermis along with extracellular matrix components (ECM): elastin and glycosaminoglycans (GAGs) such as hyaluronates and dermatan sulphate which help draw in moisture and maintain skin barrier function and healthy appearance [2]. In general, fiber‐forming collagen molecules are triple helices composed of three‐intertwined α‐helices that assemble with other collagen molecules to form collagen fibrils and fibers [2]. Fiber formation is dependent on collagen interaction with elastin and GAGs which promotes fiber formation and deposition in the skin [2].

**FIGURE 1:**
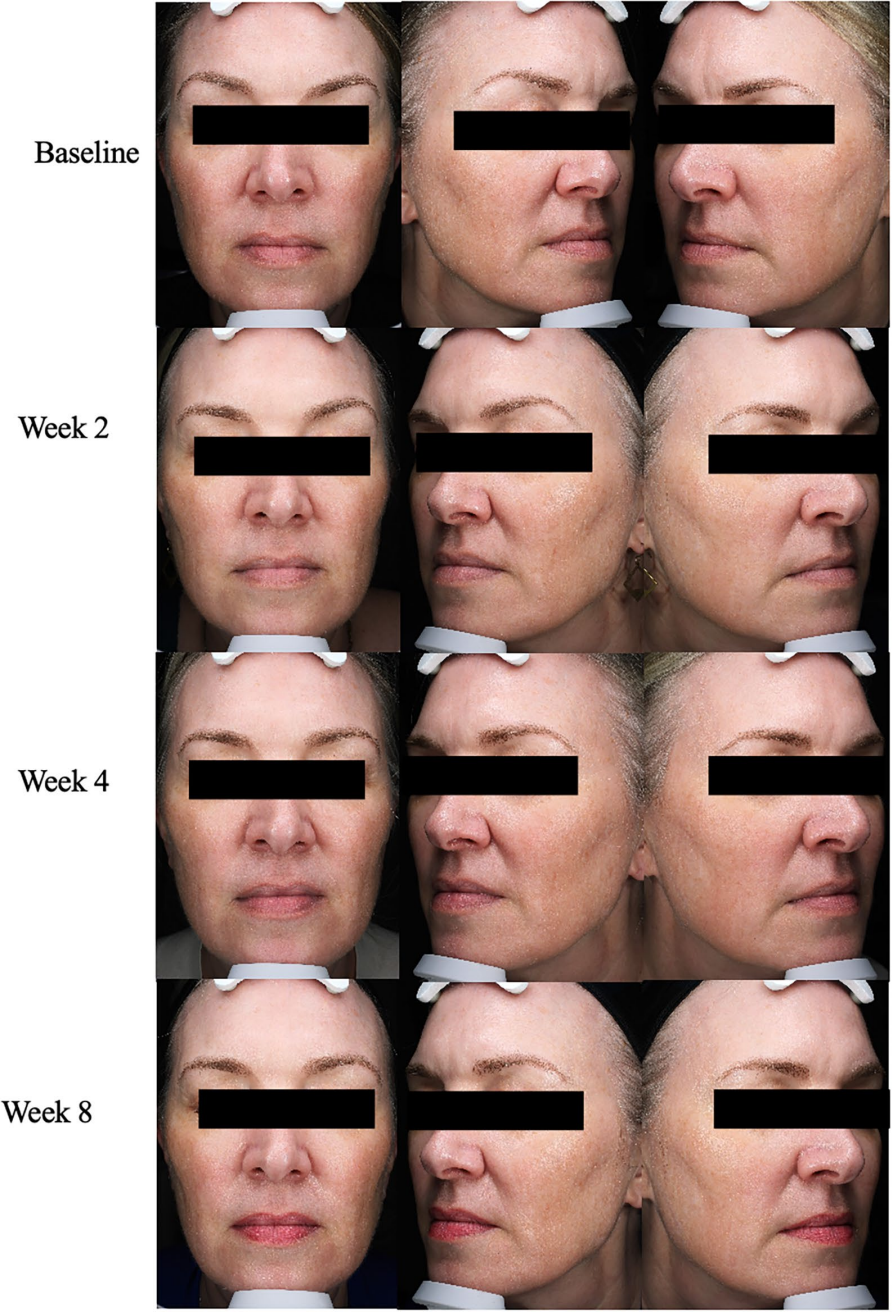
Case 1. 52‐year‐old female (FST 2). Radiesse biostimulator injections in conjunction with daily A.G.E. interrupter serum application.

Physiological aging can be characterized by a decline in fibroblast activity and subsequent decreased collagen, GAG, and elastin production. Iriyama et al. demonstrate reduced gene expression levels of collagen genes, COL1A1, COL3A1, and COL5A1 in fibroblasts from aged skin compared to that of young [3]. External factors such as sun exposure, tobacco smoke, and pollution as well as intrinsic factors such as genetics, hormones, and disease affect collagen production and turnover; thereby being important factors in aging [2]. These factors can also affect glycation, a physiological and pathological process that affects proteins in the ECM to form advanced glycation end (AGE) products which weaken collagen and accelerate aging [4]. Recent studies have suggested that skin aging preferentially occurs in the papillary dermis at the dermal‐epidermal junction (DEJ). Young skin contains approximately 80% type I collagen and 15% type II collagen. With age and photo‐damage, type I/III collagen becomes altered and decreased in the dermis [3]. Importantly, non‐fiber forming collagens IV, VII, and XVII that reinforce the DEJ in young skin also lose their structure and strength with age leading to skin aging (Figure 1). This is exacerbated by the declining ability (1.0%–1.5% per year) of the skin to replenish collagen resulting in age‐related fine lines and wrinkles [2].

Increasing studies have emphasized the importance of the DEJ in revitalizing skin function and appearance. The epidermal basement membrane at the DEJ is composed of type IV and type VII collagens as well as laminin‐511, laminin‐322, nidogen, and perlecan [3]. UV‐activated enzymes such as metalloproteinases (MMPs) can degrade proteins in the DEJ, leading to its breakdown. Notably, reconstructing this basement membrane has been shown to increase collagen expression at the papillary dermis above the DEJ [3]. Further, collagen 17A1 has been specifically linked to epidermal aging as it is downregulated by UV‐irradiation and positively correlated with impaired regeneration of keratinocytes [5]. In aged skin, collagen 17A1 is also reduced at the DEJ, which is associated with thinner skin [5]. Tissue engineering studies reveal that mimicking the DEJ and replenishing its components such as COLXVII can be important in anti‐aging strategies [6]. Peptide complexes that stimulated expression of collagen XVIII, laminin, and nidogen in the DEJ appeared to have beneficial, anti‐wrinkle effects in a small cohort study [7].

The A.G.E. Interrupter Ultra serum offers multiple benefits, notably firming and tightening skin in four key areas across the face and neck. This is achieved through a specially formulated blend of patented ingredients designed to stimulate collagen production and reduce the effects of glycation, a process that can weaken the skin's protective barrier. The serum contains: proxylane, wild fruit flavonoids, rhamnose, and gentiana lutea root extract. The proxylane and wild fruit flavonoids studies show stimulation on structural collagens and GAG as well as preventing and reversing glycation, respectively. To boost elasticity, the product contains rhamnose and gentiana lutea root extract. Rhamnose stimulates collagen IV, VII, XVII to improve DEJ anchoring while gentiana stimulates collagen I and elastin production while providing anti‐inflammatory effects (Table 1) [8]. Here, we present a real‐world case series that evaluates the use of the A.G.E. serum in a diverse patient group under real‐world conditions by dermatology experts. These real‐world cases were selected to highlight A.G.E. Interrupter serum in an integrative skincare approach to skin improvement in aging skin after collagen‐stimulating procedures.

**TABLE 1:** A.G.E. interrupter serum ingredients.

Ingredient	Action
Proxylane	Stimulates structural collagens and glycosaminoglycans
Wild fruit flavonoids	Prevents and reverses glycation
Rhamnose	Stimulates collagen IV, VII, XVII and improves DEJ anchoring
*Gentiana Lutea* root extract	Stimulates production of collagen I and elastin

## 2 Methods

### 2.1 Aim of the Project

This real‐world case series was composed to highlight integrated skincare regimens with the A.G.E. Interrupter Ultra serum. The cases demonstrate how expert dermatologists use the A.G.E. interrupter serum as an adjunct to collagen‐stimulating procedures such as Radiesse bio‐stimulation or non‐ablative and ablative laser treatments. Expert panelists' clinical reasoning and rationale are detailed in the following patient cases to serve as a guide for cosmetic dermatologists seeking to apply integrated skincare practices in their patients.

### 2.2 Steps in the Process

The real‐world cases were compiled and selected in the following steps: (1) project definition and expert panel selection, (2) data collection and preparation of patient cases, (3) patient case discussion and selection for publication, (4) literature review to support selected cases, (5) drafting, review, and finalization of the manuscript.

### 2.3 Role of the Panel

The selected expert panel consisted of 8 internationally recognized, licensed dermatologists extensive experience in cosmetic and facial rejuvenation procedures. Panelists represented clinical practices in 8 different countries including Canada, France, Luxembourg, United Kingdom, Netherlands, United States, Denmark, and Austria. Panelists were selected based on their experience in treating diverse patient populations to provide a greater understanding of integrated skincare practices in a wider variety of patients and skin types. Panelists met on September 25th, 2024, in Amsterdam, Netherlands during the European Academy of Dermatology and Venereology conference to share and discuss patient cases using the A.G.E. Interrupter serum in combination with collagen‐stimulating procedures.

The panel used the following template to gather insight through a case‐based approach:

1. Cosmetic evaluation and alignment of treatment goals.
2. Collagen‐stimulating procedure selection and treatment plan.
3. Post‐procedure recovery with A.G.E. interrupter serum.
4. Physician clinical assessment.
5. Patient self‐assessment.
6. Special considerations and key takeaways.

Each panelist shared two cases for a total of 16 cases presented during the meeting. With input from the panel, the chair of the meeting, Peter Bjerring, decided on the final 7 cases to be presented. The cases that were chosen best represented use of A.G.E. interrupter serum as a part of a collagen‐stimulating treatment plan amongst a variety of patients.

### 2.4 Collagen‐Stimulating Integrated Skincare Regimen

After each collagen‐stimulating procedure, dermatologists discussed post‐procedure skincare regimens to maximize results. The A.G.E. interrupter serum was offered to patients, and patients were instructed to apply the serum to the full face once a day for 8 weeks. At this time, all patients were supplied with A.G.E. Interrupter Serum (SkinCeuticals, USA). Healthcare providers used their discretion to apply product post‐procedure as they would in real‐world practice. Most patients applied the serum immediately after the procedure, while others waited until the night of the procedure. Patients were instructed to apply the serum to the full face once daily. Patients were encouraged to use a neutral moisturizer and broad‐spectrum sunscreen in conjunction with serum use.

### 2.5 Data Gathering and Outcome Measures

Suggested information to present included patient demographics, skin type, and collagen‐stimulating procedure selected. Patients were followed for 8 weeks with visits at baseline, week 2, week 4, and week 8. At each visit, physician assessment scores were recorded for the following categories: fine lines, wrinkles, firmness, elasticity, laxity, radiance, and smoothness. The scores were determined using the 10‐point modified Griffiths scale for photodamage (0 = none, 1–3 = mild, 4–6 = moderate, 7–9 = severe), using facial photodamage and repair as a surrogate for collagen replenishment at the DEJ [9]. Physician assessment also included a 5‐point global aesthetic improvement scale (GAIS) (1 = very much improved, 2 = much improved, 3 = improved,4 = no change, 5 = worse) and a 5‐point dryness scale (0 = none, 1 = minimal, 2 = mild, 3 = moderate, 4 = severe) at weeks 2, 4, and 8. Lastly, discomfort, tolerability, and adverse events were noted at each visit. Qualitative responses were also collected from patients at each follow‐up visit in a self‐assessment questionnaire. Patients were asked to rate 15 statements on a 5‐point Likert scale (5 = strongly agree, 4 = agree, 3 = neutral, 2 = disagree, and 1 = strongly disagree). Examples of the physician assessment and subject assessment templates can be found in Tables 2 and 3, respectively.

**TABLE 2:** Physician assessment template.

	Fine lines	Wrinkles	Firmness	Elasticity	Laxity	Radiance	Smoothness	Overall appearance	Discomfort/tolerability	Dryness	Corneometer instrumental (optional)
Panelist ID:_____________ Age:______ FITZ:____ Gender (F/M):___ Skin Type:______ Procedure:	Modified Griffith's Scale 0–9 (10 point scale) 0 = None (best possible condition) 1–3 = Mild 4–6 = Moderate 7–9 = Severe (worst possible condition)							GAIS (5 point scale: 1–5) 1 = Very Much 2 = Much Improved 3 = Improved 4 = No change 5 = Worse	Yes or No	5 point Scale: (0–4) 0 = None 1 = Minimal 2 = Mild 3 = Moderate 4 = Severe	Make/model
	Score (0–9)	Score (0–9)	Score (0–9)	Score (0–9)	Score (0–9)	Score (0–9)	Score (0–9)	Score (1–5)	Score (Yes or No)	Score (0–4)	Instrumental
Visit 1: Baseline/pre‐procedure											
Visit 1: Post‐procedure + product application											
Visit 2: Week 2											
Visit 3: Week 4											
Visit 4: Week 8											

**TABLE 3:** Subject assessment template.

	Questions	Visit 2	Visit 3	Visit 4
1	Product is suitable to use post‐Procedure			
2	Product has a pleasant texture			
3	Skin feels smoother			
4	Skin feels firmer			
5	Skin feels moisturized/hydrated			
6	Skin feels protected			
7	Skin appears tighter			
8	Skin appears renewed			
9	Skin appears younger			
10	Skin appears to have a healthy glow			
11	Visible fine lines are reduced			
12	Visible wrinkles are reduced			
13	Overall, appearance of skin is improved			
14	Overall, how satisfied are you with the product			
15	I feel more ready to do another procedure			

*Note:* 5‐point scale: 5 = strongly agree; 4 = agree; 3 = neutral; 2 = disagree; 1 = strongly disagree.

## 3 Results

Seven patient cases were selected to illustrate the use of the A.G.E. interrupter serum in conjunction with collagen‐stimulating procedures to optimize patient outcomes and satisfaction. The cases are summarized in Table 4.

**TABLE 4:** Summary of selected patient cases.

Case	Sex	Age	Fitzpatrick skin type	Concerns	Procedure	Application
1	F	52	2	Dull/dry	Radiesse	Immediately
2	F	45	3	Aging and skin laxity	Zaffiro (RM)	Day of Procedure
3	M	40	6	Skin laxity_Loss of skin firmness	Exion RFMN	Immediately
4	F	53	4	Concerns of decreased skin radiance	Exion RFMN	Day of Procedure
5	F	37	2	Finelines and loss of skin smoothness	Radiesse	Immediate
6	F	42	3	Finelines and loss of firmness	Ulterapy	Day of Procedure
7	F	67	2	Facial rejuvenation + Face lifting	Fractional picosecond	Day of Procedure

### 3.1 Case 1. Radiesse Calcium Hydroxyapatite Injections With A.G.E. Interrupter Serum

A 52‐year‐old woman, Fitzpatrick Skin Type (FST) 2, presented with concerns of dry and dull skin. In this case, the expert dermatologist felt that her concern could be most optimally treated with a collagen‐stimulating procedure such as biostimulator injections. Radiesse, a dermal filler containing calcium hydroxyapatite, was used to boost collagen production in this patient. At baseline, the patient had moderate fine lines and wrinkles with a moderate decrease in firmness, elasticity. She had severe age‐related changes in skin laxity, radiance, and smoothness. Overall, her dryness score was a 3 for moderate dryness. The patient underwent Radiesse filler injection followed by immediate application of the A.G.E. interrupter serum. Immediate post‐procedure effects included improvement in skin dryness from the nourishing serum effects and overall appearance. The patient continued to apply the A.G.E. interrupter serum daily to her whole face. By week 2, the patient saw a 1 point reduction in the severity of fine lines, wrinkles, firmness, elasticity, and laxity with 2‐point improvements in skin radiance and smoothness. The GAIS score at week 2 was a 2, much improved. At week 8, the patient continued to see improvements in firmness, elasticity, laxity, radiance, smoothness, and dryness scores while fine lines and wrinkle scores saw their maximum improvement at week 2. Final GAIS scores at week 8 revealed a very much improved appearance (Figure 1). While hoping for a lighter serum texture, the patient did not experience any discomfort or intolerance to the serum and strongly agreed that her skin felt firmer, smoother, and renewed by only week 2.

### 3.2 Case 2. Zaffiro Thermo‐Lift With A.G.E. Interrupter Serum

**FIGURE 2:**
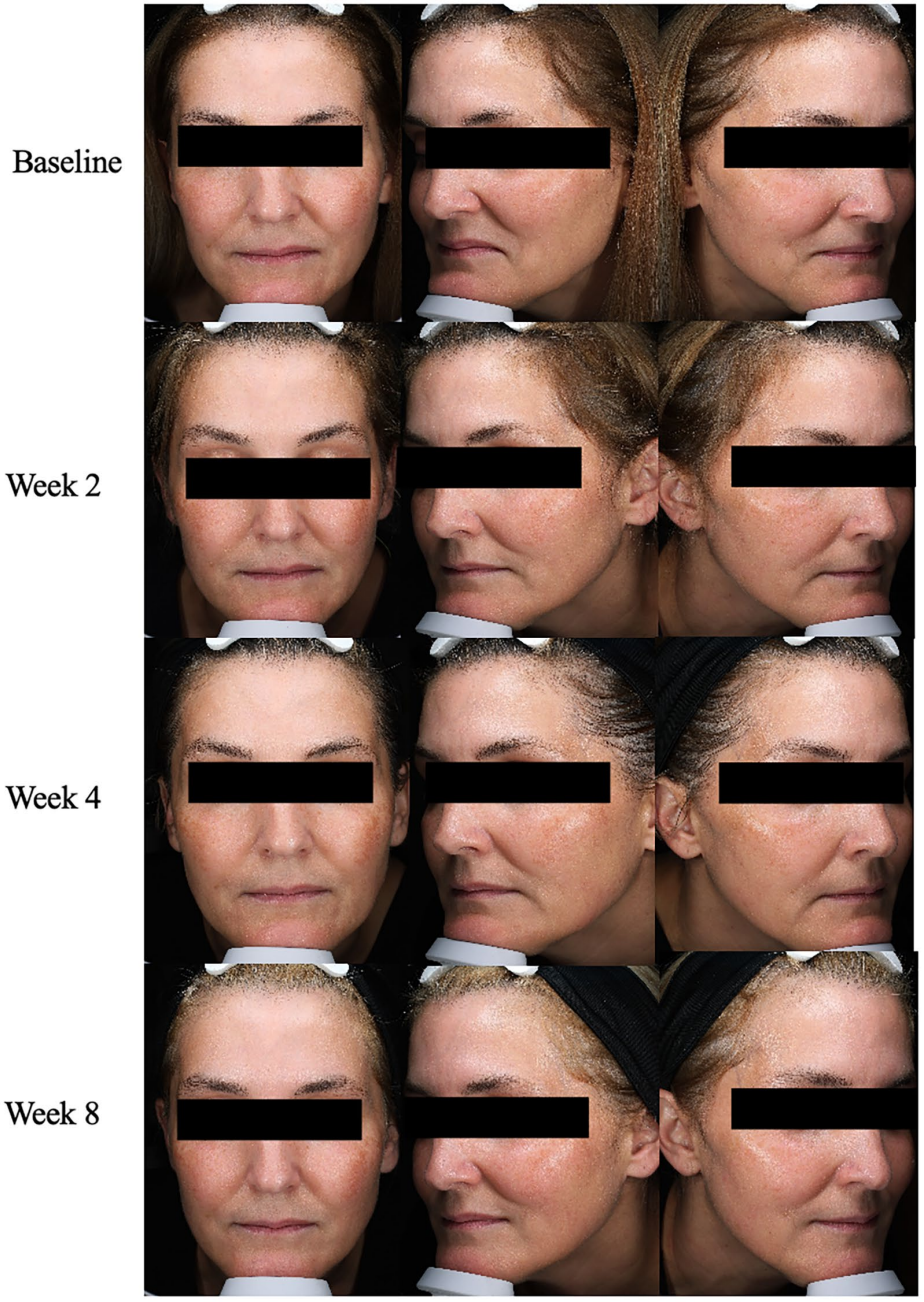
Case 2. 45‐year‐old female (FST 3). Zaffiro in conjunction with daily A.G.E. Interrupter Serum application.

A 45‐year‐old woman, FST 3, presents with concerns of aging and increased skin laxity. Zaffiro is a novel, infrared‐based collagen stimulation treatment that combines hydro‐exfoliation with infrared light to hydrate and lift the skin. Infrared technology heats the dermal layer of the skin to stimulate collagen remodeling. Zaffiro treatments occurred at baseline and week 4 and were combined with daily application of the A.G.E. interrupter serum. This combination was hypothesized to provide synergistic collagen‐boosting results. At baseline, this patient had combination skin with moderate severity scores in fine lines (5), firmness (3), elasticity (5), laxity (6), radiance (5), and smoothness (5) with minimal dryness (1) concerns. By week 2, she saw improvement in scores for fine lines, skin laxity, radiance, and smoothness; however, severity scores for wrinkles, firmness, and elasticity were unchanged. By week 8, the patient saw a reduction in severity scores to mild in fine lines (2), wrinkles (3), firmness (3), elasticity (3), laxity (3), radiance (2), and smoothness (2). GAIS score at week 8 reflected a “much improved” appearance (Figure 2). Patient self‐assessment scores reflected that the patient was highly satisfied with the product texture and skin results. She strongly agreed that by week 2 her skin felt more hydrated, protected, tighter, and younger. In all, there was a notable, steady improvement in overall appearance over the 8‐week treatment period, and the patient saw significant improvements in her skin laxity and radiance.

### 3.3 Case 3. Exion Radiofrequency With A.G.E. Interrupter Serum

**FIGURE 3:**
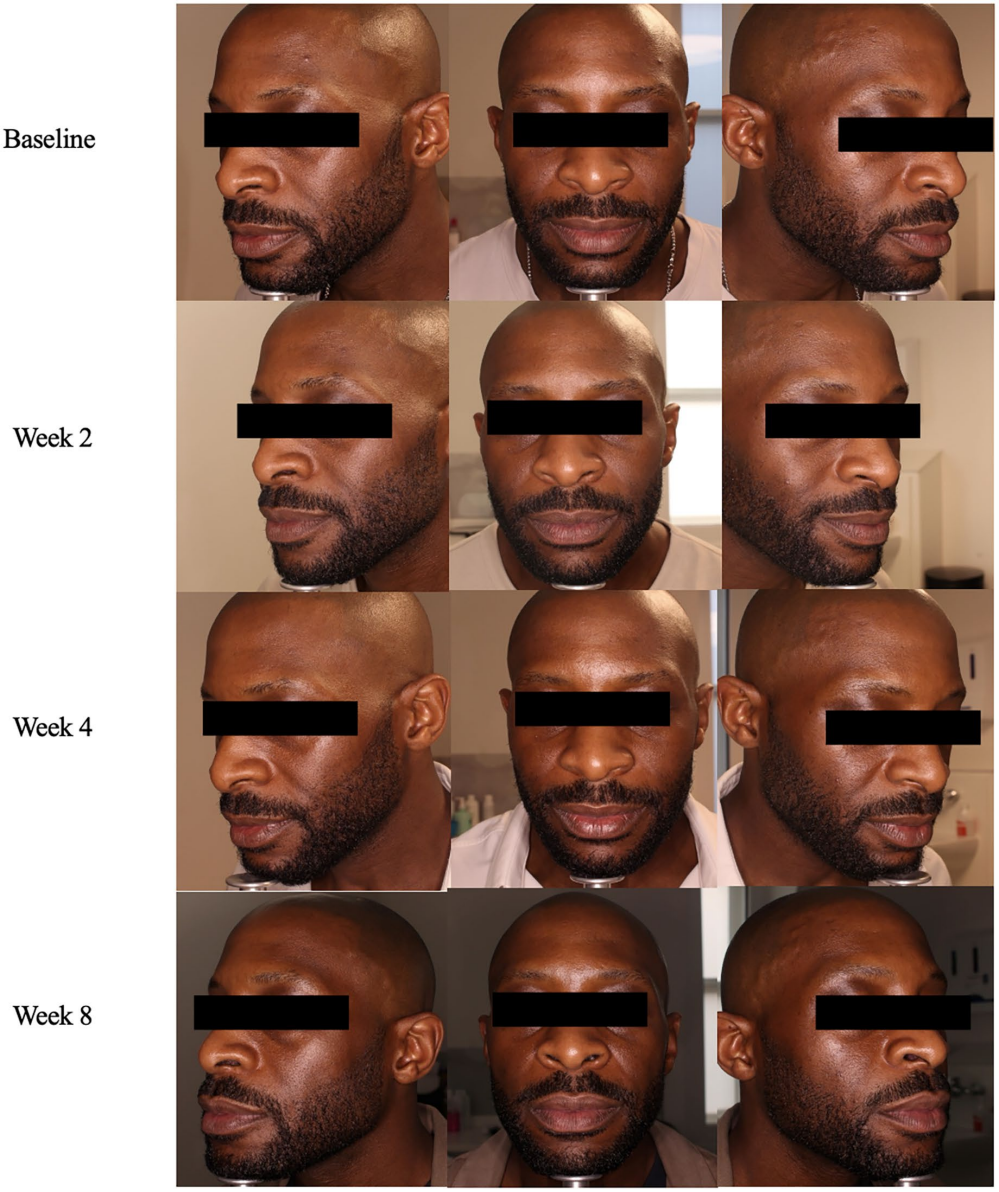
Case 3. 40‐year‐old male, FST 6. Exion radiofrequency in conjunction with daily A.G.E. interrupter serum application.

A 40‐year‐old male, FST 6, presented with concerns of skin laxity and loss of youthful skin firmness. After discussions with the expert dermatologist, the patient was treated with one session of Exion radiofrequency (RF) treatment. RF treatment paired with the daily A.G.E. interrupter serum use would optimize collagen stimulation in this patient concerned with skin rejuvenation. At baseline, the patient had moderate severity scores for fine lines (4), wrinkles (3), firmness (4), elasticity (4), laxity (5), radiance (3), and smoothness (3). Immediately after the procedure, the physician and patient did not see much of a difference in skin appearance. However, by week 8, the patient experienced a significant improvement in fine lines (2), wrinkles (2), firmness (2), elasticity (3), laxity (2), radiance (1), and smoothness (1). The physician noted that the patient's skin was much improved by the week 8 visit, GAIS score 2 (Figure 3). While the patient saw improvement in his skin, he experienced discomfort in applying the serum on day 0. While the initial texture of the serum was not favored, the patient strongly agreed that by week 8, his skin felt smoother and more hydrated.

### 3.4 Case 4. Exion Radiofrequency With A.G.E. Interrupter Serum in Patient With Rosacea

**FIGURE 4:**
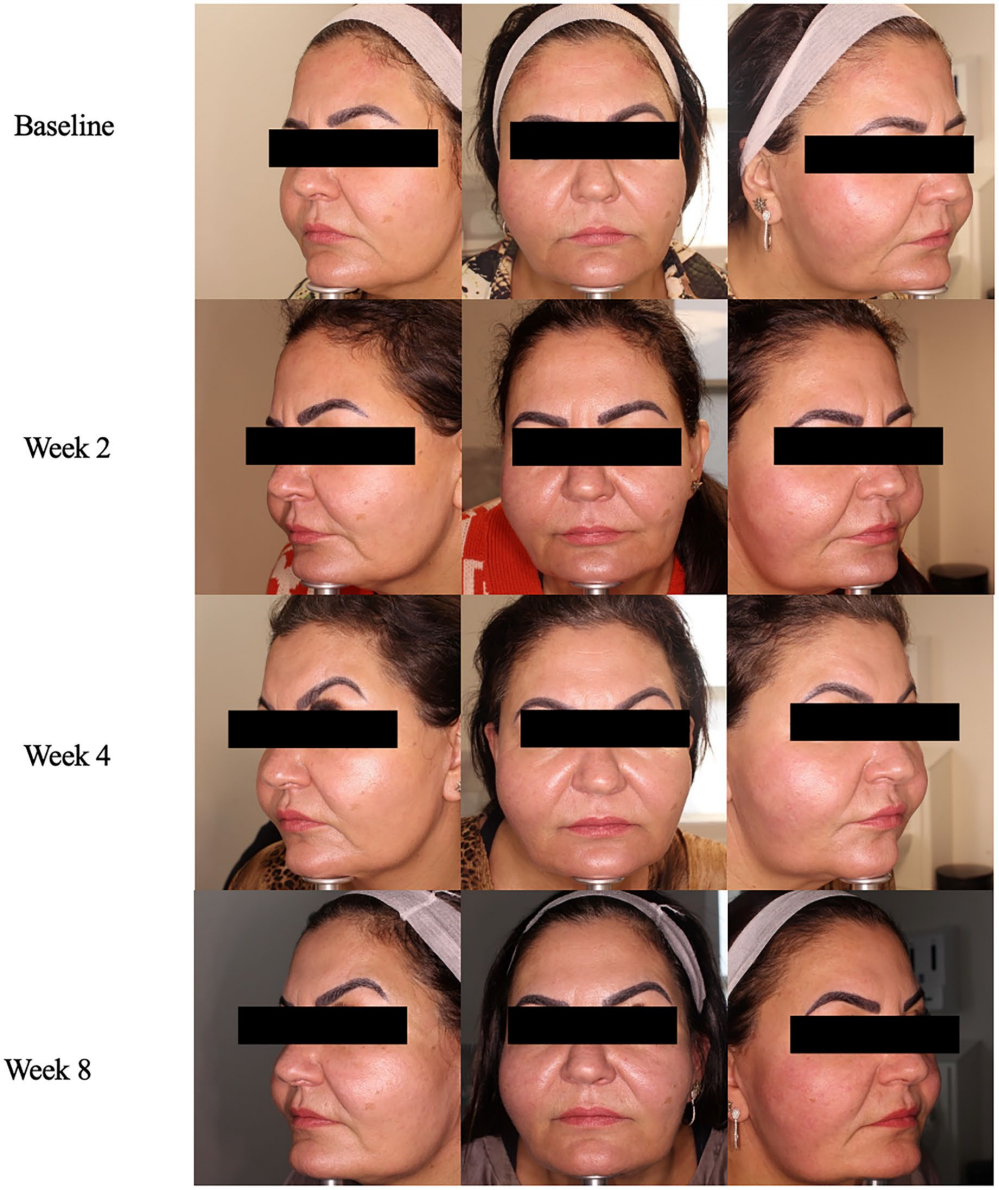
Case 4. 53‐year‐old woman, FST4, with Rosacea. Exion radiofrequency in conjunction with daily A.G.E. interrupter serum application.

A 53‐year‐old female, FST 4, presents with concerns of decreased skin radiance. She inquired about procedures that would be safe and non‐irritating to her rosacea. The physician chose a session of Exion RF with daily A.G.E. interrupter serum to help increase skin fullness and a youthful look by stimulating collagen production. At baseline, the patient had scores of 2, 2, 3, 3, 3, 4, and 3 for fine lines, wrinkles, firmness, elasticity, laxity, radiance, smoothness, respectively. The patient experienced maximum effect of the dual collagen‐stimulating approach by week 8 when she saw a decrease in severity score of her fine wrinkles (1), skin firmness (2), elasticity (2), radiance (2), and smoothness (2). By week 8, she received a GAIS score of 2, much improved by her treating physician (Figure 4). Initially, the patient did not appreciate the texture of the serum. At week 2, the patient felt that the serum was an appropriate post‐procedure treatment that made her skin feel smoother and renewed. She did not experience any adverse effects of once daily application of the serum to her whole face and did not experience any flares of her rosacea during the treatment.

### 3.5 Case 5. Radiesse Calcium Hydroxyapatite Injections With A.G.E. Interrupter Serum

**FIGURE 5:**
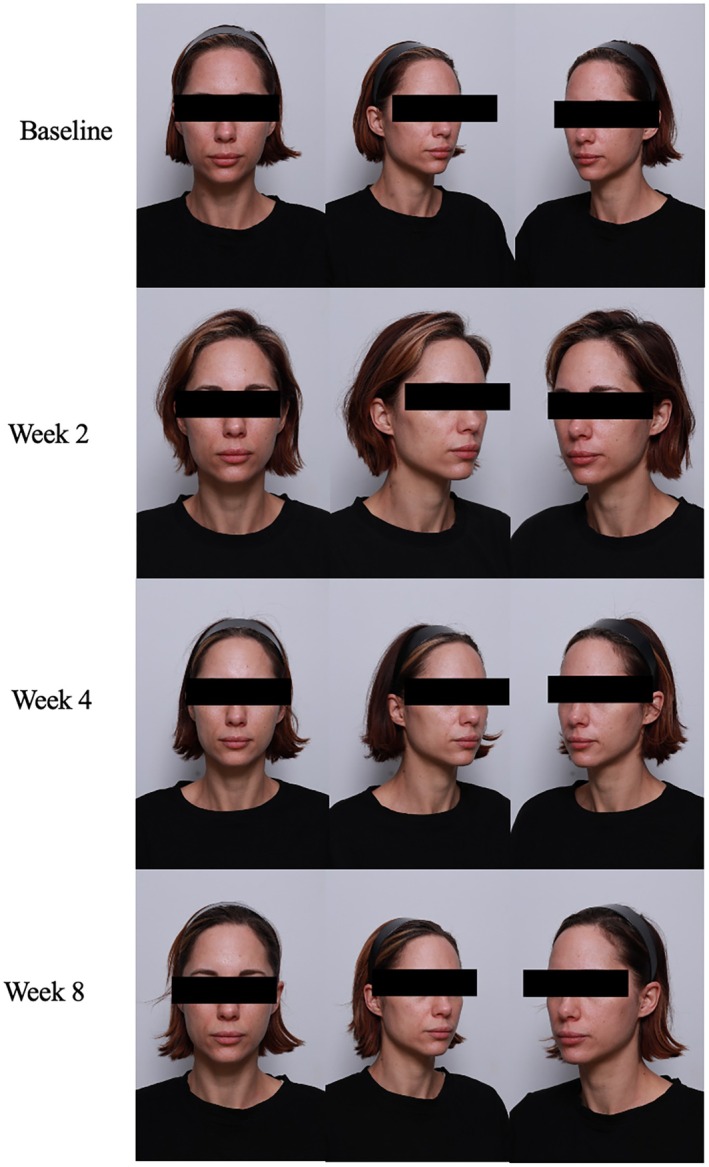
Case 5. 37‐year‐old female (FST 2). Radiesse biostimulator injections in conjunction with twice daily A.G.E. interrupter serum application.

**FIGURE 6:**
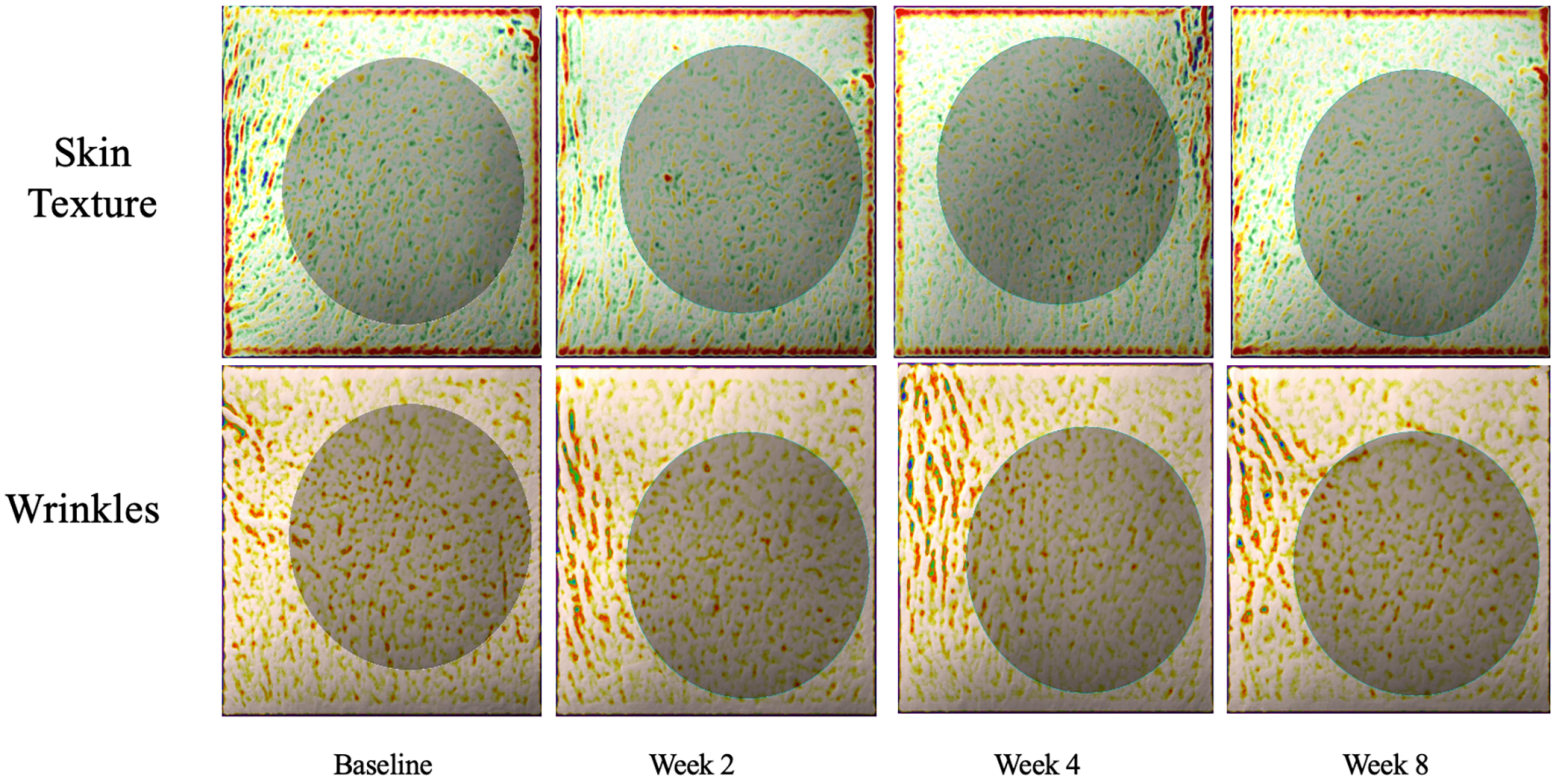
Right cheek skin texture after radiesse biostimulator injections and 8 weeks of twice daily A.G.E. interrupter serum application. Skin texture was measured using the antera 3D technology on a 37‐year‐old female (case 5).

A 37‐year‐old female, FST 2, presented with concerns of increasing appearance of fine lines and loss of skin smoothness. To treat this, the expert physician proposed a combination treatment of Radiesse dermal injections that would stimulate collagen production beneath the skin while using twice‐daily application of the A.G.E. interrupter serum to supplement collagen components from the surface of the skin. At baseline, the patient had severity scores of 4 for fine lines, 3 for wrinkles, 4 for firmness, 4 for elasticity, 3 for laxity, 4 for radiance, and 4 for skin smoothness. Moderate dryness was also noted at baseline. After 2 weeks of serum use post‐procedure, the patient felt that her skin had a healthier glow, but did not notice much difference in skin tightness or fine lines. The patient did not begin to see changes in skin qualities until week 4 when she began to see improvement in skin elasticity, laxity, radiance, and smoothness. By week 8, the score remained at 4 with no change from week 4. Her final GAIS score was 4, no change (Figure 5). Skin dryness did seem to improve; however, this improvement may have also been confounded by more intentional skincare routines during the treatment period. Interestingly, using the Delfin moisture measuring device, there was notable improvement in skin moisture in the stratum corneum over the 8‐week treatment period (Table 5). The stratum corneum saw a greater increase in moisture than the epidermis (Table 5). There was also improvement in fine lines and skin texture seen using the Antera 3D technology (Figure 6). Thus, the effect of the combination treatment may be easier to see using objective measurements rather than subjective evaluation in certain patients.

**TABLE 5:** Changes in moisture of the stratum corneum and epidermis in 37‐year‐old female (case 5).

Stratum corneum moisture	Baseline	2 weeks	4 weeks	8 weeks
Left cheek	78.4	98	96.7	88.6
Right cheek	42.8	86.2	82.3	92.2
Forehead	55.9	71.6	61.1	59.2

Epidermal moisture	Baseline	2 weeks	4 weeks	8 weeks
Left cheek	56.5	58.7	57	58
Right cheek	55.1	58.8	54.9	47.9
Forehead	60	60.4	59.6	60.3

### 3.6 Case 6. Ultherapy in Conjunction With Daily A.G.E. Interrupter Serum Application

**FIGURE 7:**
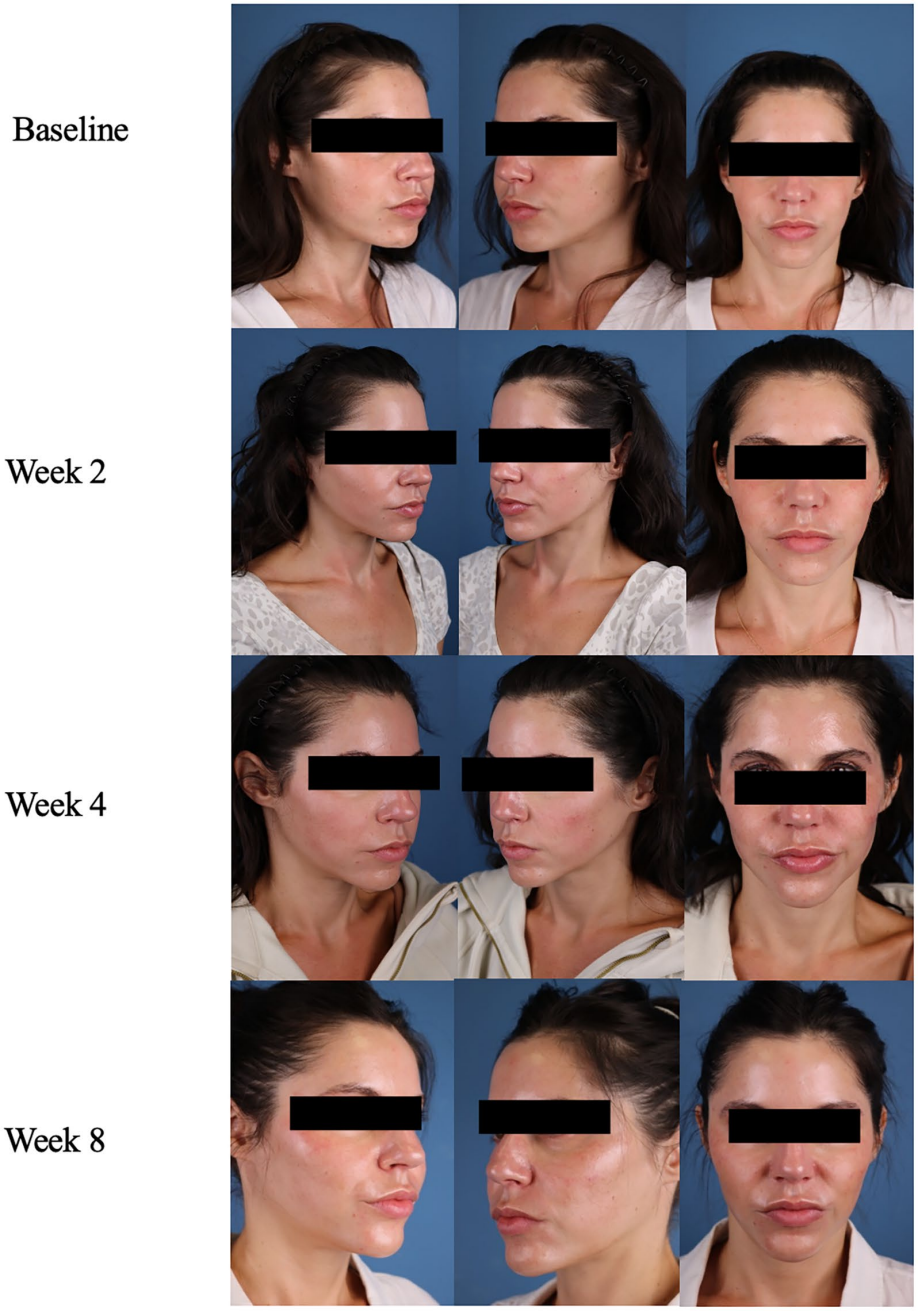
Case 6. 42‐year‐old female (FST 3). Ultherapy in conjunction with daily A.G.E. interrupter serum application.

A 42‐year‐old female, FST 3, presented to her physician's office with concerns of fine lines and loss of skin firmness. To target skin firmness and lifting, the physician chose to use Ultherapy to deliver ultrasound energy that would stimulate collagen production in the dermis. To support this effect, the patient was instructed to apply the A.G.E. interrupter serum once daily to the whole face after treatment. At baseline, the patient had the following severity scores: 7 for fine lines, 6 for wrinkles, 7 for firmness, 6 for elasticity, 5 for laxity, 6 for radiance, and 6 for smoothness. Over the next 8 weeks, the patient saw gradual improvement in her overall appearance with GAIS scores of 3 at week 2, 2 at week 4, and 1, very much improved, at week 8 (Figure 7). The patient saw the greatest improvement between week 4 and 8. Her final skin severity scores were 1 for fine lines, 2 for wrinkles, 1 for firmness, 1 for elasticity, 2 for laxity, 0 for radiance, and 0 for smoothness. The patient did not experience any discomfort or irritation with the use of the serum. She strongly agreed that the product contributed to her skin feeling smoother, firmer, tighter, and younger and felt ready for another procedure given the optimal results of this integrative skin care approach.

### 3.7 Case 7. Fractional Picosecond Laser in Conjunction With A.G.E. Interrupter Serum

**FIGURE 8:**
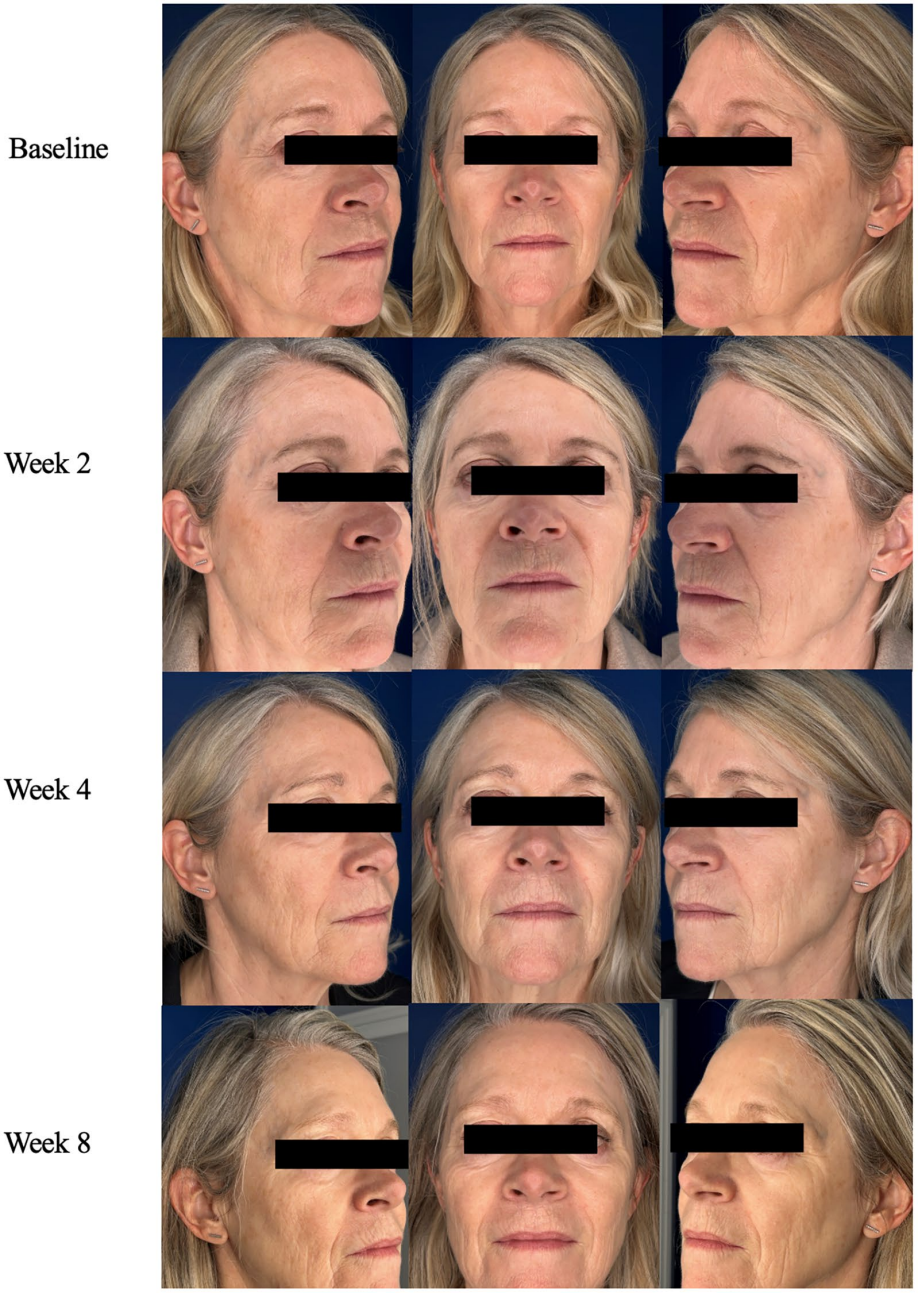
Case 7. 67‐year‐old female (FST 2). Ultherapy in conjunction with daily A.G.E. interrupter serum application.

**FIGURE 9:**
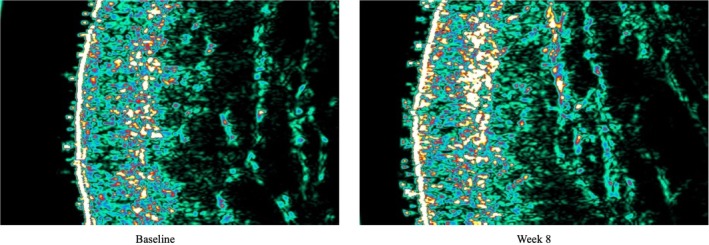
Ultrasound evaluation of dermal collagen density. The dermal collagen density was evaluated after 8 weeks of daily A.G.E. interrupter serum application and 1 session of fractional picosecond laser in a 67‐year‐old woman (case 7).

A 67‐year‐old female, FST 2, presented to the clinic for facial rejuvenation and facial lifting. The expert physician devised a treatment plan using fractional picosecond laser photo‐rejuvenation treatment with daily A.G.E. Interrupter serum over an 8‐week period. The laser was the PicoWay (Candela) laser and was used with the following settings: 1064 nm, 2.30 mJ/spot over the full face for a total of 4 passes. At baseline, the patient had the following skin severity scores: 8 for fine lines, 8 for wrinkles, 7 for skin firmness, 7 for elasticity, 8 for laxity, 6 for radiance, and 7 for smoothness. She also had mild skin dryness. Two weeks post‐laser and daily A.G.E. interrupter serum application, the patient saw improvement in severity scores across all skin quality categories. By week 8, the patient had significantly improved skin with the following severity scores: 3 for fine wrinkles, 3 for wrinkles, 4 for firmness, 3 for elasticity, 3 for laxity, 2 for radiance, and 3 for smoothness. Her GAIS score was 1, very much improved at week 8 (Figure 8). The patient denied any discomfort with serum application and saw improvement in skin dryness at the end of the treatment period. Throughout the treatment period, the patient also had her dermal collagen density measured by ultrasound technique, which noted a 73% increase in collagen structure surface area and a 20% in reduction of sun damage and aging skin surface area (Figure 9). In addition, the level of skin hydration measured by the Cortex Technology Hydration Probe revealed 66% increase in skin hydration by week 8. Overall, the patient was highly satisfied with the A.G.E. serum and its effects on her skin.

## 4 Discussion

Integrated skincare refers to the formulation of synergistic treatment regimens that lead to long‐term clinical benefit and optimized skin health and patient satisfaction [10]. The A.G.E. Interrupter serum contains actives known to stimulate collagen and support the DEJ and skin rejuvenation at the skin's surface. In the presented cases, the serum was used in conjunction with collagen‐stimulating procedures to target facial rejuvenation at the epidermis and deeper dermal layers of the skin. When used after procedures, the serum provides synergistic collagen‐boosting effects while also providing hydrating, tightening, and soothing support to help skin recover from procedures. For example, the A.G.E. Interrupter serum contains gentiana lutea root extract, which confers soothing effects to post‐procedure skin and helps to minimize adverse effects of these procedures [10, 11]. The DEJ has been increasingly recognized as a key element of age‐related skin remodeling. The ingredients within the A.G.E. Interrupter serum have been shown to improve and help restore collagen and elastin production at the DEJ to tighten skin and counteract glycation as well as other visible signs of aging.

Throughout the case series, patient and expert dermatologists agreed that the serum was effective in maximizing patient results specifically in reducing skin laxity and renewing skin appearance. It also helped reduce downtime after procedures, which improves patient experience and satisfaction. One expert also noted that in the future, it will be important to quantify the effects of the adjunct serum using tools to measure skin density, texture, wrinkles, and hydration as presented in cases 5 and 7. Other specific skin parameters that could also be measured include firmness, skin surface evenness, skin tone evenness, and glow. However, experts agreed that the serum was safe in a wide variety of skin types and comorbid skin conditions such as rosacea as well as effective at the skin's surface with evident results in their patients over the 8‐week test period. While the serum was effective in all patients presented, the most remarkable results appeared in older patients, above the age of 50. This highlights the added effects of the A.G.E. serum to reinforce networks linked in the DEJ as the most visible effects were in older patients with most likely more degraded DEJs [12].

Future studies with the A.G.E. Interrupter serum should include placebo‐controlled studies that could better elucidate the effects of the serum compared to a placebo as an adjunctive post‐procedure treatment. In addition, it may also be interesting to study the product without the use of concomitant procedures to isolate the effect of the serum alone on skin rejuvenation. Experts also shared that adjunctive use of the serum with other procedures such as CO_2_ lasers and microneedling may also be helpful in identifying the serum's ideal synergistic use. Lastly, experts were enthusiastic about the use of artificial intelligence in helping to synthesize the outcomes of experts' patient cases to help guide the path towards future clinical research priorities.

Integrated skincare regimens allow for a personalization of medicine to a patient's procedure, skin type, and budget. The A.G.E. Interrupter serum counteracts the effects of aging by providing collagen‐stimulating while enhancing the overall patient experience after procedures. In the future, recommended integrated skincare treatment guidelines built on expert consensus or clinical studies will be invaluable guides for the future of cosmetic medicine.

## 5 Limitations

The real‐world case series presented illustrate the successful use of the A.G.E. Interrupter serum in a variety of cases in the hands of multiple expert dermatologists. However, these cases do not represent data from controlled, clinical trials. In addition, the use of concomitant therapies makes it difficult to distinguish the effects of the A.G.E. Interrupter serum versus the effects of other collagen‐boosting procedures. In the future, performing a split‐face application case series may help clinically elucidate the actual effects of the serum. Despite this, patient satisfaction with results and treatment is paramount, and this case series demonstrates satisfaction and safety with the serum across a variety of skin types. Overall, these cases seek to share expert opinion, clinical experiences, and insight on how to use the serum in clinical practice.

## 6 Conclusion

The presented real‐world cases demonstrate the use of A.G.E. Interrupter serum in conjunction with collagen‐stimulating procedures to maximize patient outcomes. The serum was used in conjunction with dermal filler injections, fractional lasers, ultrasound therapy, and infrared technologies in a variety of patient skin types and ages. Overall, expert consensus was that the serum was an appropriate and efficacious adjunctive treatment for boosting collagen in facial rejuvenation. The A.G.E. Interrupter serum was safe and did not lead to any adverse reactions in the represented patient cases. Expert dermatologists' experience with the A.G.E. Interrupter serum demonstrates that the serum is a valuable option in creating integrative skin regimens after facial rejuvenation and collagen‐boosting procedures. Future studies will focus on quantifying the benefit of optimized skincare for post‐procedure care and maximizing patient results.

## Author Contributions

All authors participated in the conceptualization, design, and data gathering for this manuscript. All authors contributed to the writing and review of this manuscript. All authors contributed to the cases and development of the manuscript, reviewed it, and agreed with its content.

## Conflicts of Interest

The authors declare no conflicts of interest.

## Data Availability

The data that support the findings of this study are available on request from the corresponding author. The data are not publicly available due to privacy or ethical restrictions.
